# High-Frequency Repetitive Magnetic Stimulation Enhances the Expression of Brain-Derived Neurotrophic Factor Through Activation of Ca^2+^–Calmodulin-Dependent Protein Kinase II–cAMP-Response Element-Binding Protein Pathway

**DOI:** 10.3389/fneur.2018.00285

**Published:** 2018-05-07

**Authors:** Ahreum Baek, Eun Jee Park, Soo Yeon Kim, Bae-Geun Nam, Ji Hyun Kim, Sang Woo Jun, Sung Hoon Kim, Sung-Rae Cho

**Affiliations:** ^1^Department of Rehabilitation Medicine, Yonsei University Wonju College of Medicine, Wonju, South Korea; ^2^Department and Research Institute of Rehabilitation Medicine, Yonsei University College of Medicine, Seoul, South Korea; ^3^Department of Rehabilitation Medicine, The Graduate School Yonsei University Wonju College of Medicine, Wonju, South Korea; ^4^Department of Medicine, Yonsei University Wonju College of Medicine, Wonju, South Korea; ^5^Graduate Program of NanoScience and Technology, Yonsei University, Seoul, South Korea; ^6^Department of Biomedical Clinical Engineering, Yonsei University Wonju College of Medicine, Wonju, South Korea; ^7^Brain Korea 21 PLUS Project for Medical Science, Yonsei University, Seoul, South Korea; ^8^Yonsei Stem Cell Center, Avison Biomedical Research Center, Yonsei University College of Medicine, Seoul, South Korea; ^9^Rehabilitation Institute of Neuromuscular Disease, Yonsei University College of Medicine, Seoul, South Korea

**Keywords:** repetitive magnetic stimulation, low-frequency, high-frequency, Ca^2+^–calmodulin-dependent protein kinase II–cAMP-response element-binding protein pathway, brain-derived neurotrophic factor, Neuro-2a cells

## Abstract

Repetitive transcranial magnetic stimulation (rTMS) can be used in various neurological disorders. However, neurobiological mechanism of rTMS is not well known. Therefore, in this study, we examined the global gene expression patterns depending on different frequencies of repetitive magnetic stimulation (rMS) in both undifferentiated and differentiated Neuro-2a cells to generate a comprehensive view of the biological mechanisms. The Neuro-2a cells were randomly divided into three groups—the sham (no active stimulation) group, the low-frequency (0.5 Hz stimulation) group, and high-frequency (10 Hz stimulation) group—and were stimulated 10 min for 3 days. The low- and high-frequency groups of rMS on Neuro-2a cells were characterized by transcriptome array. Differentially expressed genes were analyzed using the Database of Annotation Visualization and Integrated Discovery program, which yielded a Kyoto Encyclopedia of Genes and Genomes pathway. Amphetamine addiction pathway, circadian entrainment pathway, long-term potentiation (LTP) pathway, neurotrophin signaling pathway, prolactin signaling pathway, and cholinergic synapse pathway were significantly enriched in high-frequency group compared with low-frequency group. Among these pathways, LTP pathway is relevant to rMS, thus the genes that were involved in LTP pathway were validated by quantitative real-time polymerase chain reaction and western blotting. The expression of glutamate ionotropic receptor *N*-methyl d-aspartate 1, calmodulin-dependent protein kinase II (CaMKII) δ, and CaMKIIα was increased, and the expression of CaMKIIγ was decreased in high-frequency group. These genes can activate the calcium (Ca^2+^)–CaMKII–cAMP-response element-binding protein (CREB) pathway. Furthermore, high-frequency rMS induced phosphorylation of CREB, brain-derived neurotrophic factor (BDNF) transcription *via* activation of Ca^2+^–CaMKII–CREB pathway. In conclusion, high-frequency rMS enhances the expression of BDNF by activating Ca^2+^–CaMKII–CREB pathway in the Neuro-2a cells. These findings may help clarify further therapeutic mechanisms of rTMS.

## Introduction

Transcranial magnetic stimulation (TMS) is a non-invasive tool that allows electrical stimulation of the nervous system and could be an ideal treatment tool due to its ability to modify brain plasticity (1). TMS can generate an electric current in the central nervous system by making short 100 μs biphasic electromagnetic pulse (2, 3). When given at regular frequencies, it is termed repetitive transcranial magnetic stimulation (rTMS) (3).

Several studies reported that changes in rTMS frequency and stimulation patterns resulted in varying long-term effects (4, 5). High-frequency stimulation (>3 Hz) stimulated cortical excitability and generally resulted in an effect that share similar aspects with long-term potentiation (LTP). In comparision, low frequency stimulation (≤1 Hz) reduced cortical excitability and induced a reduction in synaptic efficiency which were similar to long-term depression (4, 5). Various stimulation parameters such as intensity, frequency, overall patterns of stimulation, and periods determine the functional effects of rTMS on cortical excitability (6, 7). However, the neural mechanisms related with various stimulation parameters of rTMS remain unclear.

rTMS is a safe, painless, and non-invasive brain stimulation method that has been recently gaining focus as a neurorehabilitation tool with therapeutic ability (8). rTMS has been used in various neurological diseases to provide relief and reduce chronic pain (9–13). Motor symptoms in patients with Parkinson’s disease and dystonia can be ameliorated by high-frequency rTMS treatment (14–16). In stroke patients, high-frequency rTMS can increase ipsilesional cortical excitability to improve paretic limb function (17–19). Also, high-frequency rTMS may be a promising effective and safe modality in frontal cortex for Alzheimer’s disease (20). Furthermore, in amyotrophic lateral sclerosis, the brain-derived neurotrophic factor (BDNF) production may play a role by regulating with neuronal activity by rTMS in primary motor cortex (21). However, the precise therapeutic mechanisms of rTMS are still unknown.

In this study, we aimed to investigate the global gene expression patterns depending on different frequencies of repetitive magnetic stimulation (rMS) in both undifferentiated and differentiated Neuro-2a cells with multiple properties of neurons (22–25) to provide a comprehensive view of the neurobiological mechanisms. Achieving our goals, transcriptome analysis, to quantify the expression levels of individual transcripts, and possible comparison (26, 27), were conducted to compare the effect of high-frequency and low-frequency rMS in the Neuro-2a cells. Differentially expressed genes (DEGs) of high-frequency compared with low-frequency rMS were analyzed with bioinformatics tool to identify relevant cellular signaling pathways and examine the expression level to elucidate the neurobiological mechanisms.

## Materials and Methods

### Cell Cultures

**Figure 1:**
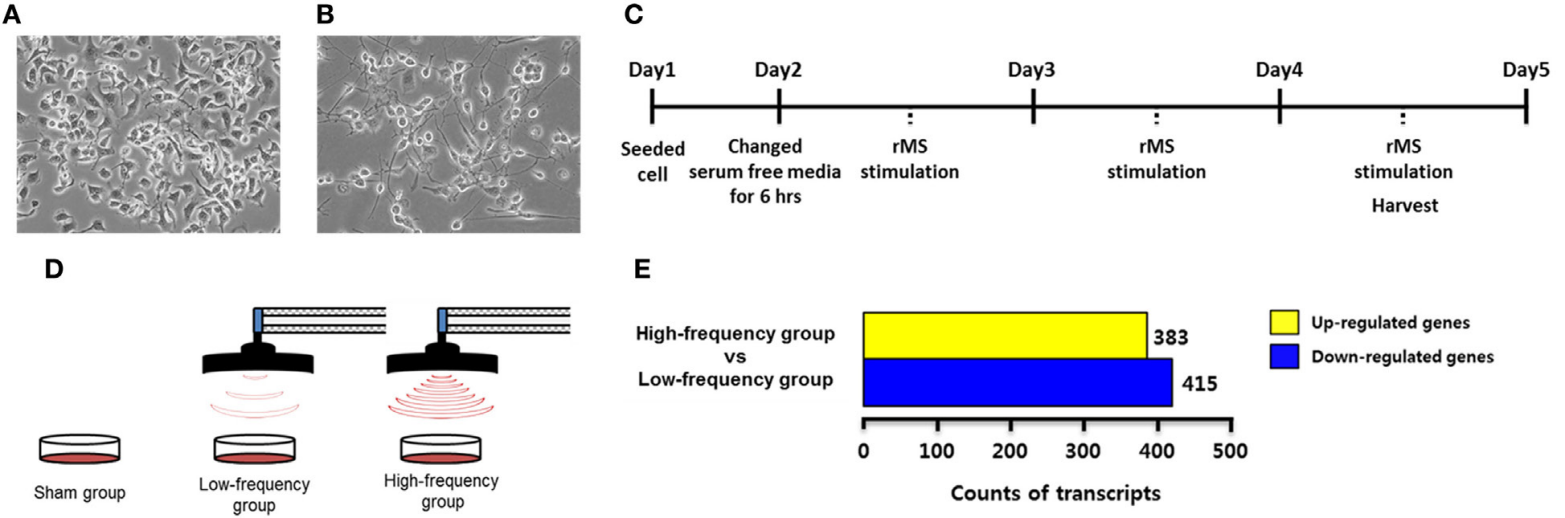
Experimental design and transcriptome analysis. **(A)** Undifferentiated Neuro-2a cells. **(B)** Neuro-2a cells were differentiated for 4 days with 2% fetal bovine serum and retinoic acid in Dulbecco’s Modified Eagle Medium. **(C)** A timeline of the experimental procedures. **(D)** A scheme of repetitive magnetic stimulation (rMS) treatment in Neuro-2a cells. The cultured cells were divided into the sham group, the low-frequency group, and the high-frequency groups and were each stimulated over 3 days. **(E)** Bar graphs show the number of differentially expressed genes with fold change ≥ |1.5| in the high-frequency group compared with the low-frequency group.

Neuro-2a cells were obtained from American Type Culture Collection (Manassas, VA, USA). Neuro-2a cells were maintained in Dulbecco’s Modified Eagle Medium (DMEM; Hyclone, Logan, UT, USA) with 10% fetal bovine serum (FBS; Serum Source International, Charlotte, NC, USA) and 1% penicillin–streptomycin solution (Gibco, Rockville, MD, USA) in a humidified atmosphere with 5% CO_2_ and 95% air at 37°C (Figure 1A). It has been reported that Neuro-2a cells are differentiated by retinoic acid (RA) treatment (28–30). According to our previous study (25), differentiated Neuro-2a cells were maintained in DMEM with 2% FBS and 20 µM of RA for 4 days in a humidified atmosphere with 5% CO_2_ and 95% air at 37°C (Figure 1B). Cells were observed under microscope and photographed using a Nikon Eclipse TS100 microscope (Nikon, Melville, NY, USA). Cells were harvested at 80% confluence using 0.25% trypsin–EDTA (Gibco). Cells were seeded on new plates and the growth medium was replaced every 2–3 days.

### Repetitive Magnetic Stimulation

In each experiment, Neuro-2a cells were rendered quiescent for 6 h by the addition of DMEM without FBS in a humidified atmosphere with 5% CO_2_ and 95% air at 37°C. Then, the cells were replaced by the growth medium and stimulated with customized rMS (Bicon-1000Pro, Mcube Technology, Seoul, Korea) as indicated in our previous studies (3, 25). To clarify the design of the experiment, the distance between the center of the magnetic coil (70 mm diameter) and the culture dish was approximately 1.0 cm. Cultured cells were divided into three groups (*N* = 5 dishes/group) as follows: the sham group (exposed to rMS but no active stimulation for 10 min), the low-frequency group (0.5 Hz stimulation for 10 min), and the high-frequency group (10 Hz stimulation for 10 min). All groups were stimulated over the course of 3 days for a duration of 10 min/day. After 3 days of stimulation, cells were harvested with 0.25% trypsin–EDTA (Gibco) as described earlier. The experimental scheme is shown in Figures 1C,D.

### RNA Isolation

By using TRIzol reagent (Invitrogen Life Technologies, Carlsbad, CA, USA), RNA was isolated from the cell pellets by following the manufacturer’s instructions. The Nanodrop spectrophotometer (Thermo Fisher Scientific, Waltham, MA, USA) was used to confirm the quality and quantity of isolated RNA.

### Transcriptome Array and Data Analysis

RNA sequencing between high-frequency group and low-frequency group in Neuro-2a cells was performed by Macrogen Inc. (Seoul, Korea) to provide a comparison. The procedures have been detailed previously (31–34).

The lists of significant differentially expressed genes (DEGs) for the high-frequency group compared with low-frequency group were submitted to the Database for Annotation, Visualization, and Integrated Discovery (DAVID v6.8; http://david.abcc.ncifcrf.gov/) *via* the Kyoto Encyclopedia of Genes and Genomes (KEGG) pathways analysis with fold change ≥ |1.5|.

### Quantitative Real-Time Reverse Transcription Polymerase Chain Reaction (qRT-PCR)

To validate transcriptome analysis results, qRT-PCR was conducted with the sham groups in Neuro-2a cells as control. Following the manufacturer’s instruction for ReverTra Ace^®^ qPCR RT Master Mix with gDNA Remover (Toyobo, Osaka, Japan), RNA were reverse-transcribed into cDNA. In a StepOnePlus Real-Time PCR System (Applied Biosystems, Foster City, CA, USA), the mRNA expression for genes of interest was validated with qPCRBIO SyGreen Mix Hi-ROX (PCR BIOSYSTEMS, London, UK). Gene expression analysis was conducted by the 2^−ΔΔCt^ method (35). Primers used for qRT-PCR are listed in Table 1.

**Table 1:** Primers used for qRT-PCR.

Gene symbol	Forward primer (5′ → 3′)	Reverse primer (5′ → 3′)
*GRIN1*	CAG GAT CGT CAG GCA AGA CA	CCA AGC AAC TGA GGG TCC TT
*CaMKII*δ	TGC ACC TGG TAG GGG ACG AT	GAA TAC AGG GTG GCT TGA TGG GT
*CaMKII*α	TGC TGC TCT TTC TCA CGC TG	TCA ATG GTG GTG TTG GTG CT
*CaMKII*γ	TTG TGC GTC TCC ATG ACA GT	TGT CAT GCT GGT GGA TGT GG
*BDNF*	GGG TCA CAG CGG CAG ATA AA	ATT GCG AGT TCC AGT GCC TT
*GAPDH*	CAT CAC TGC CAC CCA GAA GAC TG	ATG CCA GTG AGC TTC CCG TTC AG

*GRIN1, glutamate receptor ionotropic N-methyl-d-aspartate 1; CaMKIIδ, calcium/calmodulin-dependent protein kinase type II subunit delta; CaMKIIα, calcium/calmodulin-dependent protein kinase II alpha; CaMKIIγ, calcium/calmodulin-dependent protein kinase II gamma; BDNF, brain-derived neurotrophic factor; GAPDH, glyceraldehyde-3-phosphate dehydrogenase*.

### Western Blot Analysis

To confirm the protein expression of calmodulin-dependent protein kinase II (CaMKII), phospho-cAMP response element binding protein (p-CREB), brain-derived neurotrophic factor (BDNF), and ACTIN, western blot was conducted with the sham group in Neuro-2a cells. To isolated total protein, cell pellets were homogenized and dissolved using radioimmunoprecipitation assay buffer (Thermo Scientific) containing protease and phosphatase inhibitors (Abcam, Cambridge, MA, USA). Total proteins were quantified by the Quick Start^TM^ Bradford 1× Dye Reagent (BIO-RAD, Hercules, CA, USA). The samples were denatured and separated by 4–12% Bis–Tris gels (Invitrogen, Eugene, OR, USA) in 1× NuPage MES SDS Running Buffer (Invitrogen). Proteins were transferred onto a polyvinylidene difluoride membrane (Invitrogen) by 20% methanol (Merck, Darmstadt, Germany) in NuPage Transfer Buffer (Invitrogen). Membranes were blocked and then incubated overnight at 4°C with anti-CaMKII (1:1,000 dilution, Abcam), anti-p-CREB (1:1,000 dilution, Santa Cruz Biotechnology), anti-BDNF (1:1,000 dilution, Abcam), and anti-ACTIN (1:5,000 dilution, Santacruz) antibodies. The next day, blots were washed three times with 1× TBS plus Tween 20 (Biosesang, Sungnam, Korea) and incubated at room temperature for 1 h with horseradish peroxidase-conjugated secondary antibodies (1:4,000 dilution, Santa Cruz). After the blots were washed three times with TBS plus Tween 20 (Biosesang), proteins were visualized with the following enhanced chemiluminescence detection systems: Amersham^TM^ ECL^TM^ Western Blotting Detection Reagent (GE Healthcare, Little Chalfont, UK) and West-Q Pico ECL solution (GenDEPOT, Houston, TX, USA). Quantification of relative protein expression using Multi Gauge (v3.0) software (Fujifilm, Tokyo, Japan).

### Statistical Analysis

All data were expressed as means ± standard error of the mean (SEM), and all experiments were repeated at least three times with three technical replicates in each group. Statistical analyses were conducted using the Statistical Package for the Social Sciences (SPSS) for Windows version 23.0, IBM Corporation (Armonk, NY, USA). Data were analyzed with one-way analysis of variance, followed by Bonferroni’s post hoc test and with statistically significant *p*-value < 0.05.

## Results

### Gene Expression Profile by Transcriptome Analysis

To identify DEGs associated with the high-frequency group compared with the low-frequency group, we conducted transcriptome analysis by RNA sequencing. A total of 21,567 transcripts were differentially expressed from the high-frequency group compared with the low-frequency group as shown in Table S1 in Supplementary Material. In the high-frequency group, 383 transcripts were 1.5-fold higher and 415 transcripts were 1.5-fold lower compared with the low-frequency group (Figure 1E; Table S2 in Supplementary Material).

### Enriched KEGG Pathway Analysis

DEGs of the high-frequency group compared with the low-frequency group were classified based on KEGG pathways using the DAVID Gene Functional Classification Tool. Statistically significant enriched KEGG pathways specific to the high-frequency group compared with the low-frequency group are presented in Table 2 (*p* < 0.01).

**Table 2:** The enriched Kyoto Encyclopedia of Genes and Genomes pathways in high-frequency group compared with low-frequency group.

Term	Count	%	*p*-Value	Genes
mmu05031: Amphetamine addiction	9	0.0092	0.0009	FOS, CAMK2G, GRIN1, CAMK2D, PRKACA, FOSB, PPP3CA, CACNA1C, CAMK2A
mmu04713: Circadian entrainment	10	0.0102	0.0027	FOS, GNGT2, CAMK2G, GRIN1, CAMK2D, PER1, PRKACA, PER3, CACNA1C, CAMK2A
**mmu04720: Long-term potentiation**	**8**	**0.0082**	**0.0036**	**RPS6KA1, CAMK2G, GRIN1, CAMK2D, PRKACA, PPP3CA, CACNA1C, CAMK2A**
mmu04722: Neurotrophin signaling pathway	11	0.0113	0.0037	PDPK1, RPS6KA1, MAPK14, CAMK2G, PIK3CD, CAMK2D, SH2B2, SH2B1, MAPK7, MAP2K7, CAMK2A
mmu04917: Prolactin signaling pathway	8	0.0082	0.0063	FOS, SOCS2, MAPK14, SOCS1, PIK3CD, JAK2, STAT1, STAT3
mmu04725: Cholinergic synapse	10	0.0102	0.0070	FOS, ACHE, GNGT2, CAMK2G, PIK3CD, CAMK2D, PRKACA, JAK2, CACNA1C, CAMK2A

*LTP pathway is relevant to rMS treatment and is shown in a bold fonts*.

*These pathways are statistically significant (*p*-value < 0.01)*.

*FOS, FBJ osteosarcoma oncogene; CAMK2G, calcium/calmodulin-dependent protein kinase II gamma; GRIN1, glutamate receptor ionotropic N-methyl-d-aspartate 1; CAMK2D, calcium/calmodulin-dependent protein kinase type II subunit delta; PRKACA, protein kinase, cAMP dependent, catalytic, alpha; FOSB, FBJ osteosarcoma oncogene B; PPP3CA, protein phosphatase 3, catalytic subunit, alpha isoform; CACNA1C, voltage-dependent L-type calcium channel subunit alpha-1C; CAMK2A, calcium/calmodulin-dependent protein kinase II alpha; GNGT2, guanine nucleotide binding protein (G protein), gamma transducing activity polypeptide 2; PER1, period circadian clock 1; PER3, period circadian protein homolog 3; RPS6KA1, ribosomal protein S6 kinase polypeptide 1; ribosomal protein S6 kinase polypeptide 1; PDPK1, 3-phosphoinositide dependent protein kinase 1; MAPK14, mitogen-activated protein kinase 14; PIK3CD, phosphatidylinositol 3-kinase catalytic delta polypeptide; SH2B2, SH2B adaptor protein 2; SH2B1, SH2B adapter protein 1 isoform 3; MAPK7, mitogen-activated protein kinase 7; MAP2K7, dual specificity mitogen-activated protein kinase kinase 7; SOCS2, suppressor of cytokine signaling 2; SOCS1, suppressor of cytokine signaling 1; JAK2, Janus kinase 2; STAT1, signal transducer and activator of transcription 1; STAT3, signal transducer and activator of transcription 3; ACHE, acetylcholinesterase precursor*.

According to the previous studies, among several pathways, mmu04720; LTP pathway was modulated by rTMS (4, 8, 36, 37). Glutamate ionotropic receptor *N*-methyl d-aspartate 1 (GRIN1), CaMKIIδ, ribosomal protein S6 kinase polypeptide 1, and CaMKIIα were significantly upregulated in the LTP pathway. In addition, CaMKIIγ, protein phosphatase 3 catalytic subunit alpha, protein kinase cAMP dependent catalytic alpha, and voltage-dependent L-type calcium channel subunit alpha-1C were significantly downregulated in the LTP pathway. Therefore, we focused on the genes that are involved in the LTP pathway.

### High-Frequency rMS Facilitates Ca^2+^–CaMKII–CREB Pathway

**Figure 2:**
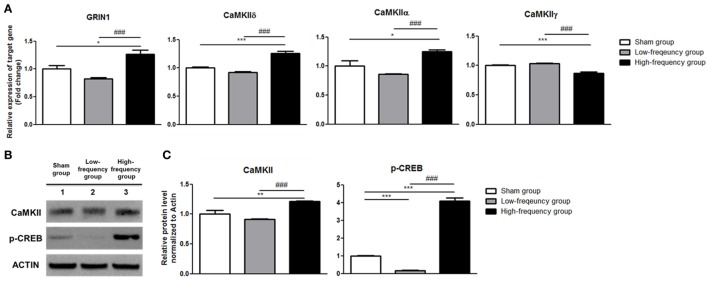
Validation of mRNA expression and protein quantification using qRT-PCR and western blot analysis in undifferentiated Neuro-2a cells. **(A)** The relative mRNA expression of target genes was normalized by sham expression and was calculated using the 2^−ΔΔCt^ method by qRT-PCR. All results are expressed as means ± SEM. **(B)** Western blot analysis was performed using antibodies against calmodulin-dependent protein kinase II (CaMKII), phospho-cAMP response element binding (p-CREB), and actin (a control). All results are expressed as means ± SEM. **(C)** Comparison of relative protein expression for CaMKII, p-CREB, and actin (a control) with Multi Guage (v3.0) software (Fujifilm). *p < 0.05, **p < 0.01, and ***p < 0.001 comparison with the sham group in undifferentiated Neuro-2a cells. ^###^*p* < 0.001 comparison with the low-frequency group in undifferentiated Neuro-2a cells.

qRT-PCR and western blot were conducted with the sham groups in Neuro-2a cells as control to validate the RNA sequencing results which identified the expression of the genes that were involved in the LTP pathway. According to the results of qRT-PCR, there were no significant changes in the low-frequency group compared with the sham group in undifferentiated Neuro-2a cells (Figure 2A). When the high-frequency group was compared with the sham group in undifferentiated Neuro-2a cells, the expression of GRIN1, CaMKIIδ, and CaMKIIα was significantly increased while CaMKIIγ expression was a significantly decreased (Figure 2A). Likewise, when the high-frequency group was compared with the low-frequency group in undifferentiated Neuro-2a cells, the expression of GRIN1, CaMKIIδ, and CaMKIIα was also significantly increased while CaMKIIγ expression was significantly decreased (Figure 2A).

**Figure 3:**
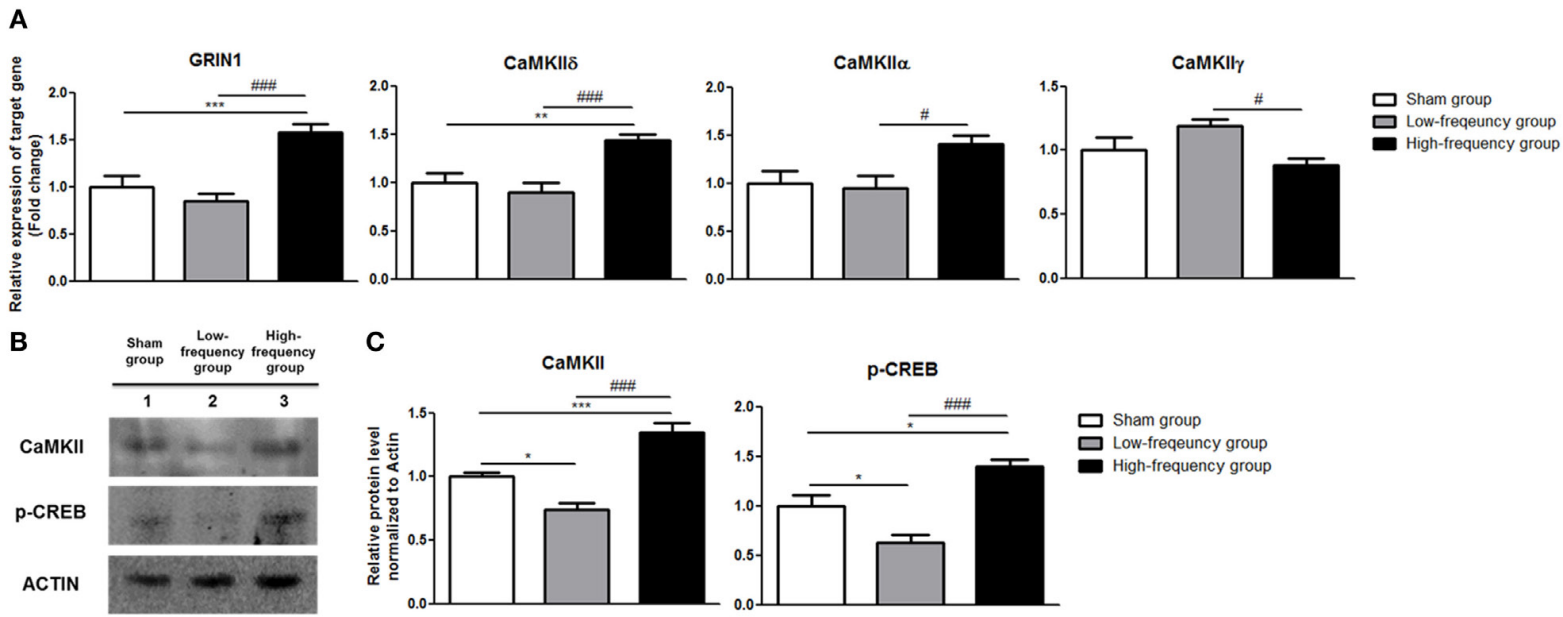
Validation of mRNA expression and protein quantification using qRT-PCR and western blot analysis in differentiated Neuro-2a cells. **(A)** The relative expression of target genes was normalized by sham expression and was calculated using the 2^−ΔΔCt^ method by qRT-PCR during neuronal differentiation of Neuro-2a cells. **(B)** Western blot analysis was performed with calmodulin-dependent protein kinase II (CaMKII), phospho-cAMP response element binding (p-CREB), and actin (as a control) antibodies in the Neuro-2a cells. **(C)** Comparison of relative protein expression for CaMKII, p-CREB, and actin (a control) in differentiated Neuro-2a cells using Multi Guage (v3.0) software (Fujifilm). All results are expressed as means ± SEM. *p < 0.05, **p < 0.01, and ***p < 0.001 comparison with the sham group in differentiated Neuro-2a cells. ^#^*p* < 0.05 and ^###^*p* < 0.001 comparison with the low-frequency group in the Neuro-2a cells.

In the same manner, there were not any significant changes in the low-frequency group compared with the sham group in differentiated Neuro-2a cells (Figure 3A). The expression of GRIN1, CaMKIIδ, and CaMKIIα was significantly increased in the high-frequency group compared with the sham group or low-frequency group in differentiated Neuro-2a cells (Figure 3A). On the other hand, CaMKIIγ expression was a significantly decreased in the high-frequency group compared with either the sham or the low-frequency group in differentiated Neuro-2a cells (Figure 3A).

GRIN1, CaMKIIδ, CaMKIIα, and CaMKIIγ are involved in Ca^2+^–CaMKII signaling pathway (38, 39). Especially CaMKII activation induced CREB phosphorylation (40, 41). We hypothesized that these genes, which were involved in Ca^2+^-CaMKII-CREB pathway, induce p-CREB by the activation of Ca^2+^–CaMKII–CREB pathway with high-frequency rMS.

Therefore, CaMKII and p-CREB protein expression levels were identified by western blot analysis. The protein expression of the CaMKII and p-CREB was significantly increased in the high-frequency group when compared with either the sham or the low-frequency group in undifferentiated Neuro-2a cells, respectively (Figures 2B,C). Furthermore, when the high-frequency group was compared with the low-frequency group in undifferentiated Neuro-2a cells, the protein expression was statistically increased (Figures 2B,C).

Likewise, the protein expression of CaMKII and p-CREB was significantly increased in the high-frequency group compared with either the sham or the low-frequency group in differentiated Neuro-2a cells, respectively (Figures 3B,C).

These data suggest that Ca^2+^–CaMKII–CREB pathway is activated by high-frequency rMS in both undifferentiated and differentiated Neuro-2a cells.

### High-Frequency rMS Facilitates BDNF Expression

**Figure 4:**
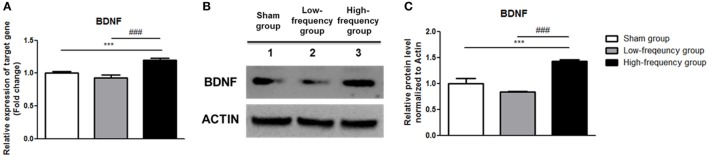
Repetitive magnetic stimulation treatment increased brain-derived neurotrophic factor (BDNF) expression in undifferentiated Neuro-2a cells. **(A)** The relative mRNA expression of BDNF was normalized by sham expression and was calculated using the 2^−ΔΔCt^ method by qRT-PCR. All results are expressed as means ± SEM. **(B)** Western blot analysis was performed using antibodies against BDNF, and actin (a control). **(C)** Comparison of relative protein expression for BDNF and actin (a control) with Multi Guage (v3.0) software (Fujifilm). All results are expressed as means ± SEM. ***p < 0.001 comparison with the sham group in undifferentiated Neuro-2a cells. ^###^*p* < 0.001 comparison with the low-frequency group in undifferentiated Neuro-2a cells.

Recently, it was reported that the Ca^2+^–CaMKII–CREB pathway plays a vital role in BDNF transcription (41, 42). Therefore, we confirmed BDNF expression by qRT-PCR and western blotting. In the low-frequency group as compared with the sham group, mRNA and protein expression of BDNF were decreased in undifferentiated Neuro-2a cells (Figure 4). However, when the high-frequency group was compared with the sham group in undifferentiated Neuro-2a cells, mRNA and protein expression of BDNF significantly increased (Figure 4). Furthermore, when the high-frequency group was compared with the low-frequency group, mRNA and protein expression of BDNF were also significantly increased in undifferentiated Neuro-2a cells (Figure 4).

**Figure 5:**
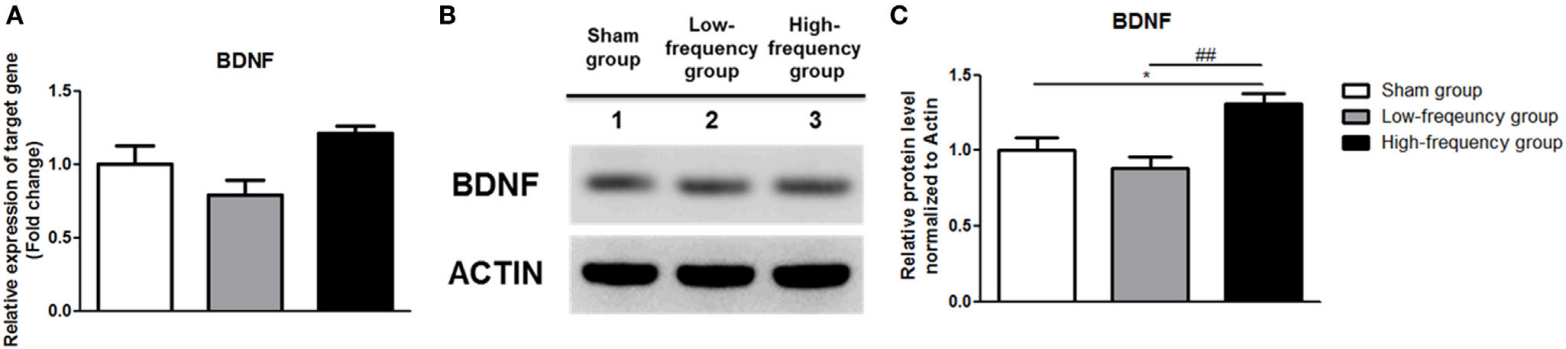
Repetitive magnetic stimulation treatment increased brain-derived neurotrophic factor (BDNF) expression in differentiated Neuro-2a cells. **(A)** The relative expression of BDNF was normalized by sham expression and was calculated using the 2^−ΔΔCt^ method by qRT-PCR during neuronal differentiation of Neuro-2a cells. **(B)** Western blot analysis was performed using BDNF, and actin (as a control) antibodies in the Neuro-2a cells. **(C)** Comparison of relative protein expression for BDNF and actin (a control) in differentiated Neuro-2a cells with Multi Guage (v3.0) software (Fujifilm). All results are expressed as means ± SEM. ***p < 0.001 comparison with the sham group in differentiated Neuro-2a cells. ^###^*p* < 0.001 comparison with the low-frequency group in the Neuro-2a cells.

In the same manner, the mRNA and protein expression of BDNF was significantly increased in the high-frequency group compared with either the sham or the low-frequency group in differentiated Neuro-2a cells, respectively (Figure 5).

Taken together, BDNF expression is increased by Ca^2+^–CaMKII–CREB pathway activation in both undifferentiated and differentiated Neuro-2a cells.

## Discussion

It has been established that different frequencies for rTMS techniques can produce different modulatory effects (5). In this study, we examined global gene expression patterns by different frequencies of rMS in both undifferentiated and differentiated Neuro-2a cells.

In this study, LTP pathway is a significant pathway which is enriched in high-frequency group compared with low-frequency group in undifferentiated Neuro-2a cells. With its ability to endure functional enhancement of synaptic connections, or structural modification of neuronal connectivity, LTP pathway is a critical process for learning and memory (43) and has been relevant in rTMS treatment (4, 8, 36, 37). In the previous study, LTP pathway was significantly increased in the high-frequency stimulation compared with the sham (44) and had long-lasting increase in synaptic efficiency as a result of the high-frequency stimulation of afferent fibers (36). In this study, we validated that the genes such as GRIN1, CaMKIIδ, CaMKIIα, and CaMKIIγ were involved in LTP pathway by high-frequency rMS in both undifferentiated and differentiated Neuro-2a cells.

GRINI, one of the subunits of the *N*-methyl-d-aspartate receptors (NMDARs), which were part of a large multiprotein complex (45), was upregulated in the high-frequency rMS in both undifferentiated and differentiated Neuro-2a cells. NMDARs, which are part of a large multiprotein complex, possess a large part in normal development and function, including synaptic plasticity, neural development, learning, and memory (46). NMDAR activity mediates CaMKII translocation to the postsynaptic density where it is maintained through a direct interaction with the C-terminal tail of the NMDAR complex (47). CaMKII is a calmodulin-dependent protein kinase that plays a crucial role in learning and memory by mediating a wide variety of intercellular responses (48, 49). There are four CaMKII isoform termed as CaMKIIα, CaMKIIβ, CaMKIIδ, and CaMKIIγ (50) that regulate calcium channel activity and gene expression (51–53). CaMKIIα, one of the major part of CaMKII, plays a critical role in hippocampal LTP and spatial learning (54, 55). CaMKIIγ is regarded one of the inhibitors of CaMKII functions (56). It also can regulate inhibitory synapses to lead long-term inhibitory synaptic plasticity (57). Taken together, Ca^2+^–CaMKII pathway is increased by high-frequency rMS in both undifferentiated and differentiated Neuro-2a cells.

Multiple signaling cascades are related in phosphorylation of CREB, including the activation of CaMKII. CaMKII has been implicated strongly in memory formation of various species as a key regulator of gene expression (41, 58–61). CREB cannot only be activated by various kinases through electrical activity, neurotransmitters, hormones, and neurotrophins, but also can promote the expression of many cAMP response elements (CREs) containing genes (62). In this study, we suggest that Ca^2+^–CaMKII–CREB pathway is activated by high-frequency rMS in both undifferentiated and differentiated Neuro-2a cells.

In the previous study, BDNF transcription and neurite outgrowth were increased through Ca^2+^–CaMKII–CREB pathway by ES in cultured rat postnatal dorsal root ganglion neurons (41). BDNF promotes neuronal survival through both inactivation of the elements that take role in cell death machinery and activation of the transcription factor, CREB (63). In addition, BDNF is a crucial protein which aids the development, differentiation, maintenance, and plasticity of brain function (64). It has been proven through several experiments that by enhancing the expression of glutamate neurotransmitters and BDNF, rTMS has the ability to regulate neuroplasticity (5, 65–67). In this study, BDNF expression is up-regulated *via* Ca^2+^–CaMKII–CREB pathway activation through high-frequency rMS in both undifferentiated and differentiated Neuro-2a cells.

**Figure 6:**
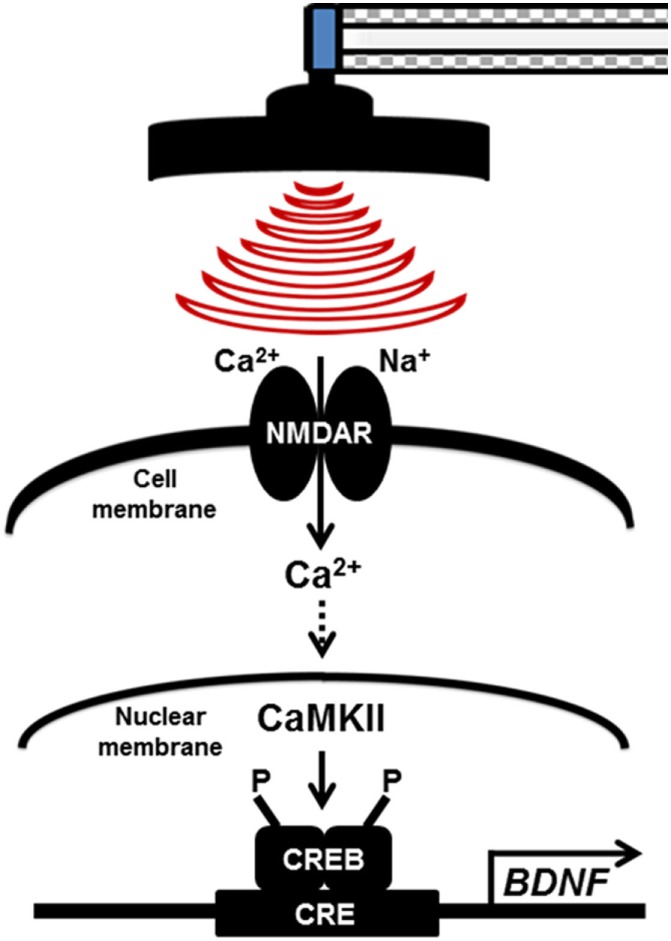
Potential therapeutic mechanisms for high-frequency repetitive magnetic stimulation (rMS) in both undifferentiated and differentiated Neuro-2a cells. The long-term potentiation pathway was confirmed to be an enriched relevant Kyoto Encyclopedia of Genes and Genomes pathway in high-frequency rMS stimulation in both undifferentiated and differentiated Neuro-2a cells. In addition, high-frequency rMS can activate the Ca^2+^–calmodulin-dependent protein kinase II (CaMKII)–cAMP-response element-binding protein (CREB) signaling pathway. In addition, phospho-CREB and brain-derived neurotrophic factor (BDNF) expression was increased *via* activation of the Ca^2+^–CaMKII–CREB signaling pathway in both undifferentiated and differentiated Neuro-2a cells. NMDAR, N-methyl-d-aspartate receptors; CaMKII, Ca^2+^–calmodulin-dependent protein kinase II; CREB, cAMP-response element-binding protein; CRE, cAMP-response element; BDNF, brain-derived neurotrophic factor.

Our finding suggests that the LTP pathway was confirmed to be a relevant enriched KEGG pathway by high-frequency rMS in Neuro-2a cells. In addition, high-frequency rMS activated the Ca^2+^–CaMKII–CREB signaling pathway, and the expression of p-CREB and BDNF was increased through the Ca^2+^–CaMKII–CREB signaling pathway in both undifferentiated and differentiated Neuro-2a cells (Figure 6).

There are several limitations in this study. Our data are focused on normal condition of undifferentiated and differentiated Neuro-2a cells, which are widely used in neurological and neurodegenerative disorders such as Alzheimer’s disease (68, 69), Parkinson’s disease (70, 71), and stroke (25, 72, 73). Therefore, in the further investigation, work on applying the mechanism, proved through this study, in various disease models to provide a cure for neurological and neurodegenerative disorders will be done. In addition to the application on various disease models, we plan to clarify the accurate therapeutic mechanism of rTMS through the application of this mechanism *in vivo* and on induced pluripotent stem cell/embryonic stem cells in the further studies to be done.

The findings in this study will provide a better understanding of the neurobiological mechanisms of neuroplasticity, through the usage of different frequencies of rTMS, and a basic information for clinical application. It will provide a comprehensive view of further therapeutic mechanisms of rTMS.

## Author Contributions

AB contributed to study conception and design, collection, and/or assembly of data and manuscript writing. EJP contributed to collect and/or assembly of data and manuscript writing. SYK contributed to manuscript writing and English editing. B-GN, JHK, and SWJ contributed to collect and/or assembly of data. SHK and S-RC contributed to data analysis and interpretation, manuscript writing, and project supervision. All the authors read and approved the manuscript.

## Conflict of Interest Statement

The authors declare that the research was conducted in the absence of any commercial or financial relationships that could be construed as a potential conflict of interest.

## Supplementary Material

Table S1 Raw data for 21,567 differentially expressed transcripts.

Table S2 Raw data for 798 differentially expressed transcripts.
